# Dr. S. S. Satchwell

**Published:** 1872-09

**Authors:** 


### PART III.

### Editorials, Correspondence and Miscellaneous.

### DR. S. S. SATCHWELL.

We give our readers, in this number, a lengthy article from the above named gentleman. Our readers will remember that, about the first of the year we published an address delivered by Dr. Norcom, before the North Carolina Medical Society. This address was published by request of one of our friends of Virginia. Dr. Satchwell took issue with Dr. Norcom, and as we had published Dr. Norcom's address, some of the friends of Dr. Satchw ll thought we ought, in justice to the latter gentleman, publish his reply—which we have now done.

Both of our North Carolina friends are men of ability and scientific attainments, and we think scientific discussion, like this, can always be made the means of doing good to the profession—particularly when due regard to feelings and language are kept in view and employed. Having published both sides our readers can form their own conclusions as to the correctness of each of our friends, touching the subject under discussion.

